# A comparison of ice wrap and subacromial injection for postoperative pain and edema control following arthroscopic rotator cuff repair

**DOI:** 10.1186/s10195-020-00556-6

**Published:** 2020-09-02

**Authors:** Yavuz Selim Kara, Onur Hapa, Yağmur Işın, Ali İhsan Kılıç, Hasan Havitçioğlu

**Affiliations:** Department of Orthopedic Surgery, Dokuz Eylül University, Izmir, Turkey

**Keywords:** Rotator, Cuff tear, Arthroscopic repair, Pain

## Abstract

**Background:**

Postoperative pain and edema are the most common problems associated with arthroscopic rotator cuff repair. The purpose of the present study was to compare ice wrap and subacromial injection (SI) as treatments for early postop pain and edema control and to contrast them with a control group.

**Materials and methods:**

59 patients treated with arthroscopic rotator cuff repair were randomized into three groups: 23 patients who received an ice wrap, 20 patients who received a SI, and a control group of 16 patients.

**Results:**

Patient demographics, comorbidities, tear retraction, degree of fatty muscle degeneration, surgical procedures, and amount of irrigation fluid were similar for the three groups, which also showed similar results regarding postoperative pain and edema control as well as analgesic consumption.

**Conclusions:**

The present study failed to show any difference in effectiveness between the two most common pain management modalities, or between those modalities and the control group.

**Level of evidence:**

IV, prospective observational study.

## Introduction

Arthroscopic rotator cuff repair is an attractive option for patients who have a symptomatic rotator cuff tear but fail to respond to conservative treatment. However, it is still associated with severe postoperative pain and decreased patient comfort [1]. Pain management is crucial to ensuring rapid rehabilitation and early discharge following the procedure [2]. Numerous pain management modalities have been described, including opioid analgesics, interscalene brachial plexus nerve block, suprascapular nerve block, periarticular analgesic injection, cryotherapy, and ice wrap [1, 3–6]. Among these, cryotherapy seems to be associated with less morbidity than the other modalities and appears to be effective at reducing pain compared to control groups [7, 8]. However, one recent study showed no difference between cryotherapy and ice wrap for pain and edema management in the early postoperative period. In that particular study, it was also reported that ice wrap is more cost-effective than compressive cryotherapy [6].

Although interscalene nerve block (INB) is reported to be one of the most effective methods of controlling pain [9, 10], it is also associated with serious systemic or local complications, including nerve injuries [1, 11–14]. One recent study reported that periarticular injection yielded better analgesia at 24 h postoperatively than INB, with fewer side effects such as nausea and arm numbness [3]. However, there does not seem to be any report of a study comparing ice wrap with subacromial injection for postoperative pain treatment and edema control after arthroscopic rotator cuff tear repair.

Therefore, the purpose of the present study was to compare ice wrap and subacromial injection as treatments for early postop pain and edema control in patients receiving arthroscopic rotator cuff tear repair, and to evaluate the effectiveness of these treatments compared to a control group. The hypothesis was that injection would be better for pain control, whereas an ice wrap would be better at reducing postoperative edema.

## Materials and methods

Patients who underwent arthroscopic rotator cuff repair (ARCR) surgery between May 2017 and July 2019 were included prospectively in the present study. Institutional ethics committee approval was received in July 2019. All the patients included in the study were operated on under general anesthesia by the same senior surgeon (O.H.) at Dokuz Eylül University, İzmir, Turkey. Of the 59 patients who were eligible for this study, 23 had an ice wrap applied, 20 patients received a SI, and the other 16 patients were designated a control group (they received neither treatment).

All patients were operated on in the lateral decubitus position under standardized general anesthesia. First, diagnostic arthroscopy was carried out. When tendon ruptures were confirmed, the single- or double-row technique was performed, depending on the tear size. After that, acromioplasty or subacromial decompression was implemented if needed. When there was inflammation of the biceps tendon or a tear or instability was identified, biceps tenotomy or tenodesis was carried out. In the SI group, after closure, a single dose of 20 ml 0.5% bupivacaine and 10 ml 2% lidocaine was injected by the surgeon. In the ice wrap group, an ice wrap was applied by the surgical team. In the control group, neither of these treatments was applied.

All patients received the same rehabilitation protocol. Patients were immobilized immediately after surgery using a sling immobilizer with abduction pillar (30° abduction). The immobilization period was three weeks for all patients. Pendulum exercises were carried out on postoperative day 0. In the ice wrap group, ice was applied for 1 h after a break of 1 h on postoperative days 0–3. On days 4–7, an ice wrap was applied three times a day for a 1 h period each time. The ice wraps were stopped after 1 week.

Patient data included age, sex, comorbidities (smoking in packs/year, diabetes duration in years, shoulder pain duration), surgical procedures (supraspinatus repair, infraspinatus repair, subscapularis repair, biceps tenodesis, biceps tenotomy, acromioplasty, subacromial decompression), operative time, and the amount of irrigation fluid (0.9% NaCl) used during surgery.

In all three groups, all of the patients were assessed for postoperative pain, opioid consumption, postoperative edema, and functional outcome preoperatively and on days 0, 1, 7, 14, and 21 after surgery. A visual analog scale (VAS) was utilized preoperatively and on days 0, 1, 7, 14, and 21 after surgery to gauge pain. To monitor postoperative edema, the distance between the anterior and posterior axillary lines (measurement* a*, in cm) and the distance from the acromioclavicular joint to the lateral epicondyle (measurement* b*, in cm) were measured preoperatively and on days 0, 1, 7, 14, and 21 after surgery.

Opioid consumption was evaluated using an opioid equianalgesic chart at day 21 after surgery [15–19]. The SF-36 score was calculated for all patients 21 days after surgery to assess functional outcome. Moreover, preoperative tendon retraction size and amount of muscle fat degeneration were evaluated on MR images using the Patte and Goutallier classifications, respectively [20, 21]. Patients who had used narcotic analgesics regularly for at least 1 month before surgery were considered to have a preoperative history of opioid consumption [22].

### Statistical analysis

All analyses were carried out with SPSS^®^ v.25.0 (Statistical Packages for the Social Sciences, version 25.0; IBM Corp., Armonk, NY, USA, 2016). Descriptive statistics were given as the mean ± standard deviation ($\stackrel{-}{x}\pm sd$) or median. The normality of numerical variables was evaluated using the Shapiro–Wilk normality test and *Q*–*Q* graphs. Two-way repeated-measures multivariate analysis of general linear models was performed to compare the three groups based on variables measured repetitively preoperatively and on postoperative days 0, 1, 7, 14, and 21. Bonferroni correction was carried out when the main variables were compared. ANOVA or the Kruskal–Wallis test was performed for variables which were not repetitive. *p* < 0.05 was considered statistically significant.

## Results

The mean age in the ice wrap group was 53 ± 10 for men (*n* = 9) and 58 ± 12 for women (*n* = 14). In the SI group, the mean age was 53 ± 13 for men (*n* = 11) and 59 ± 14 (*n* = 9) for women. In the control group, the mean age was 47 ± 11 for men (*n* = 6) and 58 ± 12 (*n* = 10) for women. The groups did not differ significantly in their age and sex distributions (*p* = 0.908 *p* = 0.484, respectively).

There was no statistically significant difference between the groups in pain duration, smoking, presence of diabetes, and number of patients who regularly consumed analgesics preoperatively (*p* = 0.717, *p* = 0.072, *p* = 0.282, and *p* = 0.237, respectively). There was also no statistically significant difference between groups in the preoperative VAS score, nor in the* a* and* b* values (*p* = 0.060, *p* = 0.390, and *p* = 0.563, respectively), nor in in terms of tendon retraction and fatty muscle degeneration (*p* = 0.577 and *p* = 0.932, respectively) (Table 1).

**Table 1:** Group comparison in terms of preoperative VAS, measurement* a*, measurement* b*, tendon retraction grade, and fatty muscle degeneration

	Ice wrap	SI	Control	
	Mean ± sd	Mean ± sd	Mean ± sd	*p*
Preop VAS	5.95 ± 2.32	6.2 ± 2.33	7.56 ± 1.41	0.060
Preop measurement* a* (cm)	22.04 ± 1.09	22.05 ± 2.52	21.12 ± 2.18	0.390
Preop measurement* b* (cm)	28.21 ± 1.97	28.75 ± 2.12	28.81 ± 1.68	0.563

	Mean ± sd Median	Mean ± SD Median	Mean ± SD Median	
Tendon retraction grade	1.91 ± 0.66 2	1.71 ± 0.57 2	1.81 ± 0.65 2	0.577
Fatty muscle degeneration grade	1.22 ± 1.24 1	1.15 ± 0.81 1	1.13 ± 1.02 1	0.932

*SI* subacromial injection

There was no statistically significant difference between the groups in terms of surgical procedures, duration of surgery, and amount of fluid used during surgery (*p* = 0.276, *p* = 0.483, *p* = 0.326, *p* = 0.426, *p* = 0.189, *p* = 0.728, *p* = 0.457, *p* = 0.095, *p* = 0.095, *p* = 0.060, and *p* = 0.134, respectively) (Table 2).

**Table 2:** Group comparison in terms of surgical procedures, operative time, and amount of fluid used during surgery

	Ice wrap	SI	Control	*p*
	*n* (%)	*n* (%)	*n* (%)	
Supraspinatus repair	21 (91)	19 (95)	16 (100)	0.276
Subscapularis repair	15 (71)	9 (47)	11 (68)	0.483
Infraspinatus repair	0 (0)	2 (10)	1 (6)	0.326
Subacromial decompression	2 (1)	0 (0)	1 (0.6)	0.426
Acromioplasty	9 (39)	11 (55)	11 (68)	0.189
Biceps				
Tenotomy	3 (13)	1 (5)	2 (13)	0.095
Tenodesis	4 (17)	3 (15)	7 (43)	

	Mean ± SD	Mean ± SD	Mean ± SD	*p*
Operative time (min)	150.43 ± 40.5	153.27 ± 29.24	176.80 ± 34.39	0.060
Amount of fluid used during surgery (l)	22.13 ± 5.23	24.15 ± 5.24	25.25 ± 3.76	0.134

The mean postoperative dose of analgesic was 84 ± 22.31 g in the ice wrap group, 87.44 ± 24.30 g in the SI group, and 91.35 ± 22.4 g in the control group. No statistically significant difference in analgesic consumption between the three groups was observed for 21 days after surgery (*p* = 0.672). There was also no statistically significant difference in analgesic consumption 21 days after surgery between those who had used analgesics preoperatively and those who did not (*p* = 0.835, *p* = 0.171, and *p* = 0.080, respectively).

The SF-36 health survey has eight scales measuring eight quality-of-life domains. There was no difference between the three groups in any of the SF-36 scales (*p* = 0.05, *p* = 1, *p* = 0.745, *p* = 0.330, *p* = 0.947, *p* = 0.114, *p* = 0.885, and *p* = 0.339, respectively).

When the intra- and intergroup temporal changes in VAS scores were examined, significant improvements in the three groups were observed on the 21st postoperative day compared to the preoperative VAS score, but there was no significant difference between the three groups. Compared to the preoperative VAS score, there were significant improvements on the 14th postoperative day in the ice group, the 7th postoperative day in the injection group, and the 1st postoperative day in the control group (Table 3).

**Table 3:** Intra- and intergroup temporal variations in VAS score

	Groups			Statistics	
	Ice wrap $\stackrel{-}{x}\pm sd$	SI $\stackrel{-}{x}\pm sd$	Control $\stackrel{-}{x}\pm sd$	*F*	*p*
Preop VAS	5.96 ± 2.33	6.2 ± 2.33	7.56 ± 1.41	2.956	0.060
VAS at 0 days postop	5.26 ± 2.61	5.3 ± 2.47	6.2 ± 2.51	0.748	0.478
VAS at 1 day postop	5.39 ± 2.37	4.45 ± 2.26	5 ± 2.37	0.874	0.423
VAS at 7 days postop	4.83 ± 2.37	3.5 ± 2.04	4.81 ± 2.34	2.442	0.096
VAS at 14 days postop	3.57 ± 2.04	2.65 ± 1.69	3.81 ± 2.37	1.729	0.187
VAS at 21 days postop	2.4 ± 1,8	2.2 ± 1.77	3.2 ± 2.17	1.327	0.274
Statistics	*F* = 12.028 *p* < 0.001	*F* = 10.866 *p* < 0.001	*F* = 9.654 *p* < 0.001		
Model statistics*					
	*F*		*p*		
Group effect	2.391		0.101		
Time effect	36.587		< 0.001		
Effect of group × time interaction	1.045		0.406		

*Two-way repeated-measures variance analysis; *a*,* b*,* c*,* d*,* e* are results of inline multiple comparison test with Bonferroni correction

When measurement* a* was examined within and between the groups, a significant increase in diameter was observed in each group on postoperative day 0 as compared to the preoperative period. An insignificant decrease was observed between days 0 and 1, whereas a significant decrease was observed between the 1st and 7th days, followed by an insignificant decrease between the 7th and 14th and the 14th and 21st days postoperatively. A significant increase in measurement* b* was observed on posteroperative day 0, followed by insignificant decreases during postoperative days 1–7, 7–14, and 14–21 (Tables 4 and 5).

**Table 4:** Group comparison of intra- and intergroup temporal variations in measurement* a*

	Groups			Statistics	
	Ice wrap $\stackrel{-}{x}\pm sd$	SI $\stackrel{-}{x}\pm sd$	Ice wrap $\stackrel{-}{x}\pm sd$	*F*	*P*
Preop measurement* a* (cm)*	22.04 ± 2.1	22.05 ± 2.52	21.12 ± 2.19	0.957	0.390
Measurement* a* (cm) at 0 days postop	24.56 ± 2.14	24.6 ± 2.23	24.06 ± 2.26	0.323	0.725
Measurement* a* (cm) at 1 day postop	24.21 ± 2.19	24.17 ± 2.42	23.62 ± 2.22	0.375	0.689
Measurement* a* (cm) at 7 days postop	22.80 ± 2.16	22.7 ± 2.27	22.00 ± 2.16	0.699	0.501
Measurement* a* (cm) at 14 days postop	22.30 ± 2.11	22.2 ± 2.5	21.43 ± 2.36	0.735	0.484
Measurement* a* (cm) at 21 days postop	22.04 ± 2.09	21.9 ± 2.08	21.06 ± 2.08	1.012	0.311
Test statistics	*F* = 22.90* *p* < 0.001	*F* = 20.92* *p* < 0.001	*F* = 21.88* *p* < 0.001		
Model statistics*					
	*F*		*P*		
Group effect	0.729		0.487		
Time effect	65.125		< 0.001		
Effect of group × time interaction	0.223		0.994		

*Two-way repeated-measures variance analysis; *a*,* b*,* c* are results of inline multiple comparison test with Bonferroni correction

**Table 5:** Group comparison of intra- and intergroup temporal variations in measurement* b*

	Groups			Statistics	
	Ice wrap $\stackrel{-}{x}\pm sd$	SI $\stackrel{-}{x}\pm sd$	Ice wrap $\stackrel{-}{x}\pm sd$	*F*	*P*
Preop measurement* b* (cm)	28.2 ± 1.98	28.75 ± 2.12	28.8 ± 1.7	0.581	0.563
Measurement* b* (cm) at 0 days postop	29.9 ± 2.3	30.5 ± 2.8	30.5 ± 2.9	0.350	0.706
Measurement* b* (cm) at 1 day postop	29.57 ± 2.1	29.95 ± 2.6	29.75 ± 1.8	0.161	0.852
Measurement* b* (cm) at 7 days postop	28.78 ± 2.56	29.3 ± 2.85	29.18 ± 1.6	0.290	0.749
Measurement* b* (cm) at 14 days postop	28.17 ± 1.7	29.2 ± 2.3	29.25 ± 1.8	2.0126	0.141
Measurement* b* (cm) at 21 days postop	27.96 ± 1.97	28.8 ± 2.26	28.88 ± 1.59	1.380	0.260
Test statistics	*F* = 7.832* *p* < 0.001	*F* = 4.648* *p* < 0.001	*F* = 3.275* *p* < 0.001		
Model statistics*					
	*F*		*P*		
Group effect	0.712		0.495		
Time effect	30.052		< 0.001		
Effect of group × time interaction	50.589		0.790		

*Two-way repeated measures variance analysis; *a*,* b*,* c*,* d*,* e* are results of inline multiple comparison test with Bonferroni correction

## Discussion

ARCR is currently a popular and well-established surgical procedure with satisfactory results [23]. However, postoperative pain control remains an issue in patients undergoing ARCR or subacromial decompression. Cryotherapy, ice wrap, and subacromial injection/infusion have been used for postoperative pain control in ARCR surgery, but the effectiveness of each is still controversial [1, 3–6].

In the literature, cryotherapy is reported to have physiologic benefits ranging from decreased local edema to the utilization of blood flow in the surgical area, decreased muscle spasm, oxygen utilization, and spinal-cord-mediated reflex arcs [3, 24–26]. Although some studies [7] have found that cryotherapy yields better results for postoperative pain and localized edema, we found that there was no difference between the groups in our study in this respect. There are two possible reasons for this. First, ice wraps could be less effective for shoulder joints. Levy et al. [27] reported that no temperature changes occurred during shoulder surgery after cryotherapy. They stated that the large muscle mass and high vascularity of the shoulder and the difficulty involved in applying cryotherapy circumferentially (unlike for the ankle and knee joints) could lead to ineffectiveness, and they showed that when cold is applied, inflammation does not stop; it is only delayed.

Second, the effect of the ice wrap could be masked by other factors that affect postoperative pain after ARCR. In the literature, postoperative pain after ARCR is reported to be affected by many factors, including physicosocial and structural factors. Ravindra et al. [28] stated that physicosocial factors have stronger associations with postoperative pain after ARCR than structural factors do. Ko et al. [29] pointed out that neuropathic pain can occur after rotator cuff tears, and this can result in severe postoperative pain, especially if there is a high preoperative VAS score or a heavy smoking habit [23]. Preoperative opioid consumption was found to be another nonstructural factor that affects postoperative pain in a study based on 35,155 patient records, which is not consistent with our findings [22]. Our smaller sample size compared to that study (35,155 vs 59) could have contributed to this difference. As for structural factors, a smaller tear size and less fatty muscle degeneration were shown to cause more pain after ARCR [30]. Although the groups did not significantly differ in tendon retraction size and fatty muscle degeneration, a failure to standardize the rupture size may have affected the results for the ice group.

In the SI group, a significant improvement in the VAS score (compared to the preoperative VAS) was seen earlier than in the ice wrap group. Saito et al. [3] stated that SI had a slow analgesic effect (this effect was not felt until the day after surgery), which could cause problems when SI is used to control acute postoperative pain. Cho et al. [31] mentioned that SI had a significant analgesic effect 3 days after surgery compared to a placebo. A few studies have shown that SI does not have a long-term analgesic effect following rotator cuff surgery, and that injection of a high dose of bupivacaine can cause chondrolysis [32, 33]. Although SI gave good results in initial studies, SI had only a marginal effect compared to the placebo. This technique has declined in popularity over the last 5 years due to uncertainity about its effectiveness and improvements in peripheral nerve blockage [33]. Our findings for the SI group showed a significant improvement in VAS score in the short term, but the lack of any significant difference between the groups is consistent with previous studies.

The highest opioid consumption (91 mg), the highest preoperative VAS score, and the earliest improvement in VAS score (the first postoperative day) was seen in the control group. These observations can be explained as follows. First, intensive analgesic consumption could cause an early drop in VAS score. Second, bicep pathologies and thus bicep surgeries were more common in the control group than the other groups (30% ice wrap, 20% injection, 56% control). It has been documented that the proximal biceps can be a source of pain in elderly patients [34]. Both tenotomy and tenodesis are valuable methods of relieving pain and improving function in patients with repairable rotator cuff tears, and yield similar outcomes [35]. Third, the validity of pain scores and evaluations can be questioned. Pain is a subjective feeling that varies among individuals even when the same stimulation is applied to all of them, and it can be affected by many variables that are unrelated to the stimulus, such as anxiety, depression, and stress relating to the disability [36].

When measurement* a* was examined within and between groups, a significant increase in diameter was observed in each group compared to the preoperative period on postoperative day 0. An insignificant decrease was observed between postoperative days 0 and 1. After that, a significant decrease was observed between postoperative days 1 and 7, followed by insignificant decreases between postoperative days 7 and 14 and between days 14 and 21. For measurement* b*, a significant increase was observed at postoperative day 0, followed by insignificant decreases between postoperative days 1 and 7, days 7 and 14, and days 14 and 21.

The changes in measurements* a* and* b* may have occurred secondary to fluid extravasation. Gupta et al. [37] noted a significant increase in mid-arm diamater on day 0 after surgery, which is consistent with our findings. Carr et al. [38] stated that there were no significant changes in deltoid muscle pressure after ARCR. Because the deltoid muscle is resistant to pressure, the distance between the acromioclavicular joint and the lateral epicondyle may be reduced to preoperative values with an earlier and decrease. For this reason, it is questionable whether the difference in arm diameter (measurement* a*) is more sensitive than the change in length (measurement* b*) as a means of evaluating postoperative edema.

This study has several limitations. First, it is retrospective. In addition, tear dimensions can be standardized and neuropathic pain can be evaluated. The application of ice wraps by patients after they have been discharged is a factor that affects the standardization of ice wrap application. Only the short-term effects of SI were evaluated in this study. The patient population was homogeneous within each group. Factors that affect postoperative pain after ARCR other than ice wrap and SI were also evaluated in this study. Lastly, this is the first study to compare ice wrap and SI for postoperative pain after ARCR.

In terms of postoperative pain control after ARCR, there was no significant difference between ice wrap and SI. The latter may have exerted a positive effect on the VAS score earlier than an ice wrap, but by the end of first month, it was not having any positive effect. Furthermore, bicep pathologies can cause severe pain in patients with rotator cuff tears, and biceps tenotomy or tenodesis is a valuable option for eliminating pain and improving functional outcomes. Postoperative pain management after ARCR is still controversial, and further studies of this issue should be performed.

## Data Availability

The datasets generated and/or analyzed during the current study are not publicly available due to the high volume of data involved, but are available from the corresponding author on reasonable request.

## Abbreviations

INB: Interscalene nerve block
ARCR: Arthroscopic rotator cuff repair
SI: Subacromial injection
